# In vitro downregulated hypoxia transcriptome is associated with poor prognosis in breast cancer

**DOI:** 10.1186/s12943-017-0673-0

**Published:** 2017-06-15

**Authors:** Basel Abu-Jamous, Francesca M. Buffa, Adrian L. Harris, Asoke K. Nandi

**Affiliations:** 1 Department of Electronic and Computer Engineering, Brunel University London, Uxbridge, Middlesex, UB8 3PH UK; 2 Department of Plant Sciences, University of Oxford, Oxford, OX1 3RB UK; 3 Cancer Research UK, Department of Oncology, Weatherall Institute of Molecular Medicine, Oxford, OX3 9DS UK; 4 The Key Laboratory of Embedded Systems and Service Computing, College of Electronic and Information Engineering, Tongji University, Shanghai, Peoples, Republic of China

**Keywords:** Breast cancer, Cell lines, Hypoxia, Co-expression, Co-regulation, Genome-wide analysis, Clustering, Bioinformatics, UNCLES

## Abstract

**Background:**

Hypoxia is a characteristic of breast tumours indicating poor prognosis. Based on the assumption that those genes which are up-regulated under hypoxia in cell-lines are expected to be predictors of poor prognosis in clinical data, many signatures of poor prognosis were identified. However, it was observed that cell line data do not always concur with clinical data, and therefore conclusions from cell line analysis should be considered with caution. As many transcriptomic cell-line datasets from hypoxia related contexts are available, integrative approaches which investigate these datasets collectively, while not ignoring clinical data, are required.

**Results:**

We analyse sixteen heterogeneous breast cancer cell-line transcriptomic datasets in hypoxia-related conditions collectively by employing the unique capabilities of the method, UNCLES, which integrates clustering results from multiple datasets and can address questions that cannot be answered by existing methods. This has been demonstrated by comparison with the state-of-the-art iCluster method. From this collection of genome-wide datasets include 15,588 genes, UNCLES identified a relatively high number of genes (>1000 overall) which are consistently co-regulated over all of the datasets, and some of which are still poorly understood and represent new potential HIF targets, such as RSBN1 and KIAA0195. Two main, anti-correlated, clusters were identified; the first is enriched with MYC targets participating in growth and proliferation, while the other is enriched with HIF targets directly participating in the hypoxia response. Surprisingly, in six clinical datasets, some sub-clusters of growth genes are found consistently positively correlated with hypoxia response genes, unlike the observation in cell lines. Moreover, the ability to predict bad prognosis by a combined signature of one sub-cluster of growth genes and one sub-cluster of hypoxia-induced genes appears to be comparable and perhaps greater than that of known hypoxia signatures.

**Conclusions:**

We present a clustering approach suitable to integrate data from diverse experimental set-ups. Its application to breast cancer cell line datasets reveals new hypoxia-regulated signatures of genes which behave differently when in vitro (cell-line) data is compared with in vivo (clinical) data, and are of a prognostic value comparable or exceeding the state-of-the-art hypoxia signatures.

**Electronic supplementary material:**

The online version of this article (doi:10.1186/s12943-017-0673-0) contains supplementary material, which is available to authorized users.

## Background

Hypoxia, that is reduced levels of oxygen, is a characteristic of solid tumours, including breast cancer, as a result of poor vascularisation, increased metabolism, and high proliferative rates [1, 2]. Amongst the different breast cancer subtypes, triple-negative breast cancer (TNBC, estrogen, progesterone and HER2 negative) is the one most frequently associated with hypoxia [3, 4]. Moreover, hypoxia is associated with increased metastasis and resistance to chemotherapy and radiotherapy, leading to poorer rates of survival [5]. These observations indicate why the gene expression signature of breast cancer tumours under hypoxia has a prognostic value, and also the reasons that hypoxia is a key area for the development of targeted therapy [5–8].

As a response to hypoxia, transcriptional programmes are induced in tumour cells that produce resistance to the stress of the low-oxygen micro-environment. This hypoxia response is mediated by the stabilisation of the hypoxia inducible factor (HIF) proteins, which transcriptionally activate over 300 genes [2, 9]. The HIF complex is a heterodimer composed of an alpha subunit and a beta subunit [9–11]. Three different HIFα proteins are known, namely the ones encoded by HIF1α, HIF2α (EPAS1), and HIF3α, while the HIFβ subunit is encoded by ARNT [12]. However, the role of HIF1α and HIF2α is relatively more understood in this process than HIF3α [12]. Normal levels of oxygen provide an essential co-factor for prolyl hydroxylases to hydroxylate HIF1 and 2α. This marks them for degradation by the proteasome after being ubiquitinated by Von-Hippel Lindau (VHL) syndrome protein. Thus abundance of oxygen represents a signal for the degradation of HIF while hypoxia results in its stabilisation [9–11, 13].

HIF heterodimers bind to the hypoxia response element (HRE) motif, with the consensus sequence of RCGTG, at the promotors of many genes resulting in their transcriptional activation [2, 14]. These genes participate in key biological processes, such as angiogenesis, pH regulation, metabolism, autophagy, invasion, metastasis, and others, which promote tumour growth [2, 13, 15–18].

Targeting HIF directly has proven to be difficult [19]. However, genes activated by HIF under hypoxia, such as the key angiogenesis regulator VEGF, or the unfolded protein response, mediated by factors like XBP1 and ATF4, can be targeted [8, 20–23]. So far there has been a larger focus on genes transcriptionally activated by HIFs. However, many genes are downregulated in hypoxia indirectly regulated by HIF [24, 25], which could provide opportunities for synthetic lethality. Therefore, we have conducted an analysis extending a recently published consensus clustering method [26] and using all available experiments exposing breast cell lines to hypoxia and hypoxia related treatments, using different setups and timing. The power of our clustering approach is that it allows us to analyse these datasets collectively, to define genes consistently co-expressed in hypoxia, upregulated and downregulated, and the potential mechanisms of their regulation.

## Results and analysis

### Cell-line datasets & experimental procedures

We considered a comprehensive series of sixteen human breast cancer cell-line microarray datasets, covering different breast cancer subtypes, and testing the hypoxia response using different experimental setups (Table 1). The first column shows the unique name which is used hereinafter to refer to each of these datasets. Due to the differences in the microarray platforms used to generate these datasets, not all genes are represented by probes in all of the datasets. However, 15,588 genes were found in common in at least thirteen out of the sixteen datasets, and they represent the input set of genes to the following analysis. These genes are listed in Additional file 1.

**Table 1:** Breast cancer microarray datasets in contexts related to hypoxia

ID	GEO acc.	Year	Platform acc.	Platform	N^b^	Cell line(s)	Description	Ref.
D01	GSE3188^a^	2005	GPL2507	Sentrix Human-6	7	MCF7	Same samples of the last two datasets in the same order, but a different platform.	[67]
D02	GSE47533	2014	GPL6884	Illumina HumanWG-6 v3.0	4	MCF7	Time-series data through 48 h of exposure to hypoxia (1% O)	[40]
D03	GSE41491	2012	GPL14877	Affymetrix HG-U133+ 2.0	8	MCF7	Time-series data through 24 h of exposure to hypoxia (1% O)	[68]
D04	GSE47009	2014	GPL16686	Affymetrix HuGene 2.0 ST	3	MCF7	Samples at normoxia, hypoxia, and anoxia, respectively	-
D05	GSE18494	2009	GPL9419	Affymetrix HG-U133+ 2.0	4	MDA-MB-231	Time-series data through 12 h of exposure to hypoxia (0.5% O)	[43]
D06	GSE3188^a^	2005	GPL96	Affymetrix HG-U133A	3	MCF7	Samples at normoxia, hypoxia, and normoxia exposed to DMOG, respectively	[67]
D07	GSE17188^a^	2010	GPL6480	Agilent Whole Human Genome G4112F	3	SCP2 subline of MDA-MB-231	Time-series data through 24 h of exposure to hypoxia.	[69]
D08	GSE17188^a^	2010	GPL6480	Agilent Whole Human Genome G4112F	3	LM2 subline of MDA-MB-231	Time-series data through 24 h of exposure to hypoxia.	[69]
D09	GSE15530	2010	GPL6947	Illumina HumanHT-12 V3.0	4	MCF7	Normoxia samples versus hypoxia samples, each is either transfected with non-specific shRNA or with reptin shRNA.	[28]
D10	GSE45362	2013	GPL10558	Illumina HumanHT-12 V4.0	4	MB231RN-LM derived from MDA-MB-231	Non-transfected samples versus transfected with has-miR-18a, each is in either a control medium or treated with Cobalt(II) chloride (CoCl2) hypoxia-mimicking agent.	[70]
D11	GSE29406	2012	GPL571	Affymetrix HG-U133A 2.0	4	MCF7	Normoxia samples versus hypoxia samples, each is either untreated or treated with lactic acid.	[71]
D12	GSE18384	2010	GPL6884	Illumina HumanWG-6 v3.0	4	MCF7	Normoxia samples versus hypoxia samples, each is either non-transfected or transfected with siRNA#1 against JMJD2B	[72]
D13	GSE3188^a^	2005	GPL570	Affymetrix HG-U133+ 2.0	4	MCF7	Samples exposed to / transfected with oligogectamine, HIF1α siRNA, HIF2α siRNA, or both siRNAs, respectively. All samples were grown under hypoxia (1% O) for 16 h.	[67]
D14	GSE33438	2011	GPL1708	Agilent Whole Human Genome G4112A	4	MCF7 & ZR-75-1	Samples from each of the two cell lines were exposed to hypoxia for 24 h or 48 h	[73]
D15	GSE49953	2013	GPL570	Affymetrix HG-U133+ 2.0	4	T47D & MDA-MB-231	A control sample and an XBP1-knocked-down sample from each of the two cell lines. All samples are under hypoxia and glucose deprivation.	[74]
D16	GSE30019	2012	GPL6884	Illumina HumanWG-6 v3.0	6	MCF7	Time-series data through 24 h of reoxygenation after having been in hypoxia (0.5% O) for 24 h.	[75]

^a^Some accession numbers refer to datasets which include samples that in reality represent more than one dataset, either because they belong to different microarray platforms or to different cell lines

^b^This is either the number of time-points in time-series data or the number of conditions in other types of data

An important generalisation was made to UNCLES type A method [26, 27] in order to enable its use to cases where missing features are present (see Methods). In particular, the extended UNCLES allows inclusion of genes which are represented by probes in some rather than all datasets analysed. Had the condition of gene inclusion been as required by the original UNCLES method, described in [26], only 7714 genes would have been considered in this analysis, which is less than 50% of the array content. Therefore, this modification allows for more comprehensive analysis of available datasets and reduces the amount of data filtered out due to missing data or less complete platforms.

The UNCLES method does not perform its collective analysis to such group of heterogeneous datasets by merging them into a single dataset; rather it clusters each one of them individually by multiple clustering methods, and then finds the consensus result of these individual clustering results. In other words, UNCLES finds the consensus membership of genes in clusters based on results from independent clustering analyses of multiple datasets rather than merging the datasets themselves. This approach overcomes the problems that appear while attempting merging datasets generated by different platforms and with different conditions. This is detailed in the Methods section and in [26, 27].

Individual clustering methods employed at the first step of the UNCLES method were *k*-means with Kauffman initialisation (KA), self-organising maps (SOM) with bubble neighbourhood and four-by-four grid, and hierarchical clustering (HC) with Ward’s linkage. The experiment was repeated with the numbers of clusters (*K* values) of 8, 9, 10, 16, 18, 24, 30, and 40, and while varying the tuning parameter δ from zero to unity with steps of 0.1. The resulting clusters from all of these experiments were exposed to the M-N scatter plots [26] in order to select the top clusters (see Methods).

### Consensus clustering identifies two clusters of genes oppositely co-regulated under hypoxia across 16 diverse cell-line datasets

**Fig. 1:**
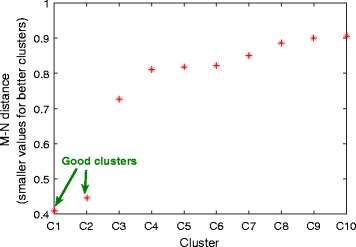
M-N scatter plots’ distances. Quality evaluation of the first ten clusters selected by the M-N scatter plots technique. Quality is quantified by the M-N distance metric, which has smaller values for better clusters. The M-N plots technique inherently orders the clusters that it selects from the best to the worst. In this Figure, it is clear that the first two clusters have significantly smaller M-N distance values than the rest, and therefore they are labelled as “good clusters” and are exclusively considered for downstream analysis

As explained in Methods, the M-N scatter plots technique is used to select the best clusters out of the pool of clusters generated under different *K* and *δ* values. This selection is done sequentially in a descendant order of the quality of the selected clusters and the quality measure is the distance of the cluster from the top-left corner of the M-N plot. This metric should be minimised, i.e. lower values of it indicate better clusters in quality. The M-N distances of the first ten clusters, labelled as C1 to C10, are plotted in Fig. 1. This Figure shows that the first two clusters have significantly lower distances, and thus better quality, than the rest of the clusters. Therefore, these two clusters were chosen for the following investigations.

**Fig. 2:**
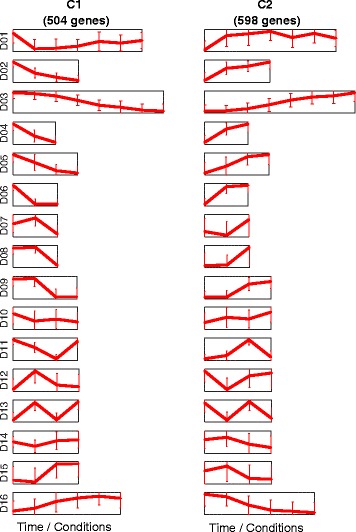
Clusters average profiles over datasets. The average profiles of the clusters C1 and C2 in each of the 16 datasets (D01 to D16). The two columns of plots correspond to the two clusters while the 16 rows correspond to the 16 datasets. The plots are scaled horizontally to reflect the number of data points (time/conditions) that each dataset has (ranging from 3 in D04, D06, D07 and D08 to 7 in D01). A detailed version of this Figure is included in Additional file 2

The first cluster, C1, includes 504 genes, while the second cluster, C2, includes 598 genes. Full lists of genes in these two clusters are provided in Additional file 1 and the average expression profiles of these two clusters in each of the sixteen datasets are plotted in Fig. 2. A fully labelled version of this Figure is provided in Additional file 2. Interestingly, it is notable in Fig. 2 that the profiles of the two clusters are consistently negatively correlated in all of the datasets. Also, the first cluster is downregulated with hypoxia while the second is upregulated in general.

**Fig. 3:**
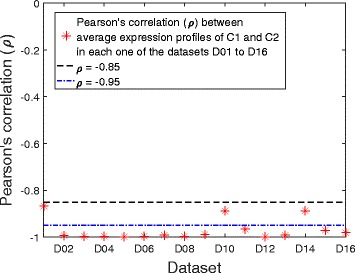
Correlation between C1 and C2. Pearson’s correlation (*ρ*) between the average profiles of C1 and C2 in each of the 16 datasets D01 to D16. Two dashed horizontal lines are shown at *ρ* = −0.85 and *ρ* = −0.95. The two clusters are clearly negatively correlated in all datasets as their correlation values never exceed −0.95 except in three datasets, which in their turn are still below *ρ* = −0.85

In order to measure the negative correlation between C1 and C2 quantitatively, Pearson’s correlation (*ρ*) was calculated between their average profiles in each of the 16 datasets and is shown in Fig. 3. The correlation between the two clusters does not exceed the very low value of −0.95 in any of the datasets except for D1 (*ρ* = −0.87), D10 (*ρ* = −0.89), and D14 (*ρ* = −0.89). This very strong and consistent negative correlation, with no phase-shift, over sixteen different datasets from a wide range of experiments suggests that these two clusters may be oppositely co-regulated by a common mechanism.

D02, D03, D05, D07, and D08 represent time-series datasets in which breast cancer cell lines were exposed to hypoxia for durations ranging from 12 to 48 h (Table 1). The first time-point in each of them is at normoxia, or in other words, at zero hours after exposure to hypoxia. It can be seen that, in all of these cases, C1 is downregulated gradually in hypoxic conditions while C2 is upregulated (Fig. 2). In contrast, the dataset D16 represents time-series through reoxygenation, i.e., the first time-point is at hypoxia (0.5% Oxygen), followed by shifting the cells back to normal oxygen levels (21%) and observing their genetic expression for 24 h following the shift (Table 1). Agreeing with the previous observation, C1 shows gradual upregulation under reoxygenation while C2 shows gradual downregulation (Fig. 2).

Similar observations can be seen in the other datasets while comparing normoxic with hypoxic conditions (Fig. 2 and Additional file 2). In some datasets, exposure to some agents mimics the effect of real hypoxia, such as exposure to 2-oxoglutarate dependent dioxygenase inhibitor dimethyloxalylglycine (DMOG) (D01 and D06), and to CoCl_2_ (D10). Similarly, normoxic effects were observed in some datasets in cell lines under hypoxia when certain hypoxia-negating modifications were applied; examples include transplanting with has-miRNA-18a (D10), treatment with lactic acid (D11), and knocking down key regulators of hypoxia such as HIF1A (D01 and D13) and XBP1 (D15).

However, some treatments which were expected to reduce the effect of hypoxia were not observed to have an effect on these two clusters. For instance, transfection with reptin siRNA in D09 or knocking down HIF2A in D01 or D13 did not show a significant reduction in the effects of hypoxia. As for reptin, it was previously shown that about 25% of genes up- or down-regulated by hypoxia are reptin-dependant while the rest are not [28]. The clusters C1 and C2 are significantly enriched with reptin-independent hypoxia-regulated genes (*p*-values: 1.7 × 10^−23^ and 1.2 × 10^−22^ for C1 and C2 respectively) but are not as significantly enriched with reptin-dependent genes (*p*-values: 6.5 × 10^−3^ and 6.6 × 10^−3^ for C1 and C2 respectively); this indeed justifies our observation here. As for knocking down HIF2A, it was in comparison with knocking down HIF1A, and it is clear that these two clusters have dependency on HIF1A rather than on HIF2A. Despite that, knocking down HIF1A could not fully restore the expression of these two clusters to the same level as in normoxia (D01).

### Clusters of co-regulated genes map to distinct pathways, biological processes and cellular components

We have performed GO term analysis over the genes in C1 and C2 to identify the most significantly enriched biological processes and cellular components in them using the GeneCodis online tool [29–31]. Full lists of results are available in Additional files 3 and 4 for C1 and C2 respectively.

**Fig. 4:**
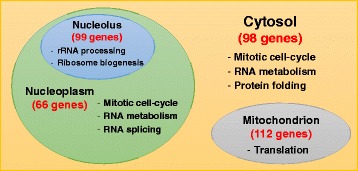
The localisation of the biological processes of the genes in C1. The Figure shows the numbers of genes included in C1 which localise into the cytosol, mitochondrion, nucleolus, and the nucleoplasm, as well as the main biological processes which are enriched in each one of these sub-cellular components

Analysis of C1 GO terms revealed four major groups of genes participating in specific biological processes with significant localisation in the nucleolus, the nucleoplasm, the mitochondrion, or the cytosol. Those are summarised in Fig. 4.

The general characteristic of C1 is that it is highly enriched with genes participating in growth-related processes such as the mitotic cell-cycle, gene expression (e.g. RNA metabolic process and RNA splicing), and protein synthesis (e.g. translation, ribosome biogenesis, rRNA processing, tRNA processing, and protein folding).

The localisation of the genes participating in these processes highlights rRNA processing and ribosome biogenesis (aka RRB) genes in the nucleolus; mitotic cell-cycle, RNA metabolism, and RNA splicing in the nucleoplasm; genes participating in mitochondrial protein translation localise, clearly, in the mitochondrion; and some cell-cycle, RNA metabolism, and protein folding genes localised in the cytosol. These observations can be summarised as four groups of growth-related processes which take place in four different locations of the cell are down-regulated simultaneously by hypoxia. Thus there is a major suppression of processes that use high amounts of ATP, potentially contributing to cell survival.

C2, which is upregulated under hypoxic conditions, is enriched with many genes well described as part of the hypoxia transcriptome and are HIF targets (Table 2). These genes include those involved in signal transduction [32], positive regulation of I-kappaB kinase/NF-kappaB cascade [33], carbohydrate metabolism and glycolysis [34–38], chromatin remodelling [39], inhibition of cell proliferation [17] (Table 2). Also, C2 included a large number of genes which participate in regulation of DNA transcription, whether that was in the form of transcription factors, chromatin organisation, or otherwise (Table 2).

**Table 2:** Highly enriched cellular processes’ and components’ GO terms in C2 (598 genes)

GO term	Back. freq.	Freq.	Corrected *p*-value
Biological processes			
Regulation of transcription; DNA-dependent	1609	80	4.4 × 10^−14^
Glycolysis	45	12	5.6 × 10^−9^
Carbohydrate metabolic process	290	23	7.3 × 10^−7^
Signal transduction	1176	51	8.2 × 10^−7^
Cell cycle	435	25	4.9 × 10^−5^
Response to hypoxia	175	14	4.2 × 10^−4^
Chromatin modification	224	15	1.5 × 10^−3^
Negative regulation of cell proliferation	341	18	4.3 × 10^−3^
Multicellular organismal development	945	33	9.3 × 10^−3^
Positive regulation of IκB kinase/NFκB cascade	140	10	9.9 × 10^−3^
Cellular components			
Nucleus	5441	230	4.1 × 10^−39^
Cytoplasm	5302	218	6.5 × 10^−35^
Cytosol	2146	93	4.0 × 10^−14^
Membrane	4065	135	4.3 × 10^−12^
Golgi apparatus	958	44	3.8 × 10^−7^
Endoplasmic reticulum	1000	45	4.0 × 10^−7^
Nucleoplasm	891	38	2.0 × 10^−5^
Plasma membrane	3575	101	2.5 × 10^−5^
Integral to membrane	4400	118	3.1 × 10^−5^
Golgi membrane	420	23	4.9 × 10^−5^
Endosome	280	18	6.7 × 10^−5^

The first column shows the GO term, the second column shows the background frequency, i.e. the number of genes belonging to this GO term amongst the 34,208 background genes in the Genecodis database, the third column shows the number of genes belonging to this term amongst the cluster’s genes, and the fourth column shows the corrected *p*-value

**Fig. 5:**
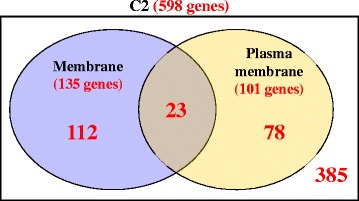
Venn diagram of C2 genes belonging to two major membrane-related GO terms. The two membrane-related GO terms in this diagram are ‘membrane’, which includes 135 genes and ‘plasma membrane’, which includes 101 genes. The number of genes in the union of the two sets is 213 genes. The large rectangle represents all of the genes in C2, which are 598 genes. The space within the rectangle but outside all of the circle represents genes included in C2 but not included in any of the two circles, and this includes 385 genes

As for the cellular components in which C2 genes localise, in addition to nuclear and cytoplasmic genes, membrane-related components are predominant. Figure 5 represents a Venn diagram showing the numbers of genes which belong to the two GO terms ‘membrane’ and ‘plasma membrane’. Also, significant numbers of C2 genes localise to the Golgi apparatus and the endoplasmic reticulum, as highlighted in Table 2.

The distribution of genes participating in different processes over those different cellular components in C2 is not as distinct as was shown in C1 (Fig. 4). In summary, the C2 genes responding to hypoxia have roles throughout the cell, such as regulation of transcription and chromatin remodelling in the nucleus, the activation of carbohydrate metabolism, including glycolysis, and signalling.

KEGG pathway analysis were performed by using the GeneCodis online tool in order to identify the pathways with genes over represented in the clusters C1 and C2 [29–31]. Full results can be found in Additional files 3 and 4 for the clusters C1 and C2, respectively. The results of this analysis shows agreement with the results of the GO term analysis. For instance, C1 is highly enriched with various growth-related and cell-cycle pathways such as RNA transport, ribosome biogenesis, pyrimidine and purine metabolism, spliceosome, cell-cycle, folate biosynthesis, and RNA polymerase; all are with corrected *p*-values lower than 1 × 10^−4^. Additionally, the proteasome pathway is strongly correlated, corrected *p*-value (1.1 × 10^−8^).

As for C2, which is up-regulated under hypoxia, carbohydrate metabolism pathways, namely glycolysis and the metabolism of fructose, mannose, amino sugars, starch, sucrose, and pentose phosphate, have corrected *p*-values lower than 1 × 10^−3^. Importantly, the renal cell carcinoma pathway is also significantly represented in the cluster with a corrected *p*-value of 1.8 × 10^−4^. This pathway includes genes that are regulated by the HIF transcription factor, which is stabilised by the mutations of VHL which are typical of this cancer type. Nine genes from the cluster C2 are listed as members of this pathway, BRAF, EGLN1, EGLN3, GAB1, MAP2K1, MET, SLC2A1, VEGFA, and VEGFC. As for the 14 genes which are assigned by the GO term analysis to the GO process term ‘response to hypoxia’, they overlap with the aforementioned nine genes, but they do not include all of them. The 14 genes are ALDOC, ANGPTL4, BNIP3, CITED2, EGLN1, EGLN3, LONP1, NOL3, PAM, PLOD1, PLOD2, SCNN1B, TH, and VEGFA. As can be seen, only three genes are in the intersection of the two groups of genes, while the union of them includes 20 distinct genes. Again, the general observation here is that KEGG pathway analysis agrees with the GO term analysis in identifying carbohydrate metabolism and response to hypoxia/HIF targets as the main groups in C2. However this also shows the current deficits in the deposited pathways resulting in discordance between databases and indicting the need for more comprehensive approaches and multiple genes for any likely utility in tumour hypoxia classification.

### Analysis of HIF targets confirms significant differences amongst upregulated genes and downregulated genes in response to hypoxia

Hypoxia-inducible factor (HIF) is a master regulator of gene expression in response to hypoxia [40]. Therefore, we have analysed the contents of the cluster C2 in light of five studies which produced lists of potential targets for HIF [12, 25, 41–43]. Those five studies produced in total seven lists of potential HIF targets as some of them produced separate lists for HIF1α and HIF2α. The seven lists, labelled as L1 to L7, are described in Table 3.

**Table 3:** Description of seven lists of genes which were identified as potential targets for HIF

List	TF	*N*	Approach	Reference
L1	HIF1α	500	Integrative genomes (computational & experimental)	[41]
L2	HIF1α	394	Genome-wide ChIP	[25]
L3	HIF2α	131	Genome-wide ChIP	[25]
L4	HIF1α	311	ChIP-chip	[43]
L5	HIF	216	Genome-wide computational approaches	[12]
L6	HIF1α	323	ChIP-seq.	[42]
L7	HIF2α	268	ChIP-seq.	[42]

The first column shows the ID of the list, the second column shows the HIF TF which was considered in the study, the third column shows the number of distinct genes included in the list (N), the fourth column states the approach adopted to produce the list, and the fifth column shows the reference including the name of the first author and the year

Strikingly, the union of all of those lists includes 1521 genes, of which 1172 were identified by our analysis, while the intersection includes two genes only, namely, RSBN1 and PPP1R3C (Table 4). This is expected due the wide difference in the experimental approaches. Interestingly, C2 is significantly enriched with such HIF target genes (145 out of 598 C2 genes) with a *p*-value of 1.2 × 10^−38^. A conservative analysis of the very tight intersection of all lists (two genes only) showed one of the two genes, RSBN1, still overlapping with C2. On the other hand, C1 is not significantly enriched with HIF targets.

**Table 4:** Different combinations of the seven lists of HIF potential targets

Combination	*N*	In this study	C1	p.v.	C2	p.v.	Comments
Union of all	1521	1172	42	0.26	145	1.2 × 10^−38^	-
Intersection of all	2	2	0		1	7.5 × 10^−2^	Gene RSBN1 in (C2)
Intersection of studies with HIF1α (L1, L2, L4, L5, and L6)	11	10	0		6	5.7 × 10^−7^	Genes in (C2): CA9, DARS, GAPDH, PLOD2, RSBN1, and SPRY1
In 3 lists or more	144	126	0		61	1.2 × 10^−52^	-
In 4 lists or more	60	49	0		30	1.5 × 10^−30^	-
In 5 lists or more	28	22	0		15	6.4 × 10^−17^	Genes in (C2): CA9, DARS, ENO1, GAPDH, GBE1, HK2, INHA, INSIG2, KIAA0195, P4HA1, PLOD2, RSBN1, SPRY1, STC2, WSB1

The first column describes the combination of the lists, the second column shows the number of genes in the combination, the third column shows the number of those genes which are included in the input 15,588 genes in our study, the fourth and the fifth columns show the numbers of those genes which are included in C1 and C2, respectively, with their *p*-values, and the last column shows some comments

The 15 genes in C2 which appear in at least five lists of HIF targets include genes which are well-known participants in response to hypoxia processes together with less well-understood genes, where according to GO term annotation, there is no specific biological process in which they are known to participate or a molecular function which they are known to perform (KIAA0195 and RSBN1). Amazingly, RSBN1 is one of the two only genes over which there is a consensus amongst all of the seven lists as a potential HIF target, not previously reported to be induced by hypoxia, and supposedly exclusive to spermatids. This is further discussed in the Discussion section.

### Cell-line-derived clusters are not conserved in clinical datasets, but form sub-clusters of highly correlated genes with distinct biological functions

Having obtained C1 and C2 by clustering gene expression in cell lines, we investigated their expression in breast cancer clinical tumours. The Cancer Genome Atlas (TCGA) is a comprehensive resource for high-throughput molecular biological data regarding cancers, and analysed expression profiles in 1026 breast cancer tumours [44]. This data measures the expression of 20,531 genes in those 1026 samples, and covers 14,493 genes out of the 15,588 genes included in our study (Additional file 5). In cell lines the cluster C1 contains 504 genes; 471 of these genes are represented and expressed in this clinical dataset. Similarly, the cluster C2 in cell lines contains 598 genes; 564 of these genes are represented and expressed in this clinical dataset (Additional file 6).

**Fig. 6:**
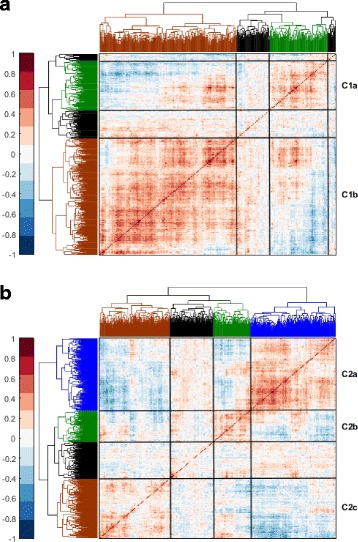
C1 and C2 form smaller sub-clusters of genes in the TCGA clinical dataset. Spearman’s autocorrelation amongst the genes in **a** C1 and **b** C2 over the 1026 breast tumour samples provided by the TCGA database. Indexing of the rows starts from top to bottom and for the columns starts from *right* to *left*, i.e. the first gene is represented by the *top-most row* and the *right-most column*. Colours in the heat map range from *dark brown* for +1.0 to *dark blue* for −1.0. Sub-clusters of genes based on this autocorrelation are labelled to the *right* of the heat maps and *white lines* are drawn over the heat maps to highlight their borders

Then, we calculated the pairwise Spearman’s autocorrelation amongst the genes which belong to each cluster, C1 and C2, in the 1026 tumours. After applying hierarchical clustering, the results are shown in the form of heat maps in Fig. 6. C1 genes form two tight sub-clusters, labelled as C1a and C1b, which include 116 and 273 genes respectively. Similarly, C2 genes form three tight sub-clusters, namely C2a, C2b, and C2c, including 204, 88, and 168 genes respectively. The lists of genes in those sub-clusters are provided in Additional file 5.

We tested whether these sub-clusters also form tight clusters in other clinical datasets and Table 5 lists the details of six clinical datasets including the analysed TCGA dataset. For each sub-cluster, the mean-square error (MSE) has been calculated in each one of the six datasets as well as for 1000 randomly generated clusters of a similar size. By fitting a normal distribution to the MSE values of the randomly generated clusters, a *p*-value has been calculated for obtaining the observed MSE value, or a more favourable one, randomly. The results revealed that the sub-clusters C1a, C1b, C2a, and C2b form significantly tight clusters in all six datasets with *p*-values ranging from 10^−6^ to smaller than 10^−300^. On the other hand, the complete cluster C2 shows relatively poor tightness in the clinical datasets compared to its sub-clusters with *p*-values ranging from 0.99 to 10^−6^ in all datasets except for the METABRIC-disc dataset in which the *p*-value of C2 is about 10^−31^. Nonetheless, it is still not as significantly correlated in that dataset as C1a, C1b, C2a, and C2b as they all have *p*-values smaller than 10^−290^ therein.

**Table 5:** Breast cancer clinical datasets

Title	Number of samples	OS and ER information?	Reference
TCGA	1026	YES	[44]
GE	196	NO	[76]
GSE2034	286	YES	[77]
GSE3494	251	YES	[78]
METABRIC-disc	997	YES	[79]
METABRIC-val	995	YES	[79]

GO term analysis (using the GeneCodis tool [29–31]) was employed to identify any specific enrichment of biological processes in those sub-clusters. Full results are shown in Additional file 5. Both C1 sub-clusters (C1a and C1b) have significant shares of the mitotic cell-cycle genes. Nonetheless, C1b is specifically highly enriched with translation, RNA metabolism, gene expression, protein folding, rRNA processing and ribosome biogenesis (RRB), and regulation of apoptotic process, all with corrected *p*-values lower than 10^−5^ in C1b and higher than 10^−3^ in C1a; such differences in *p*-values between the two sub-clusters demonstrate the specificity of enrichment of these processes in C1b.

The distinction amongst the three C2 sub-clusters is more apparent; hypoxia response is focused in the smallest of the three sub-clusters, namely C2b, which also includes some glucose metabolism and signal transduction genes; all with corrected *p*-values lower than 0.01. As for C2a, the only process which is significantly enriched in it is the regulation of DNA-templated transcription, with a corrected *p*-value of 8.4 × 10^−10^; no other process is enriched herein with a corrected *p*-value lower than 0.01. Interestingly, the two other clusters do not have this process significantly enriched despite it being the most enriched process in the mother cluster C2. As for C2c, it is enriched with many carbohydrate metabolism (including glycolysis) and signal transduction genes. This shows that, while each one of the two clusters is consistently co-expressed over 16 different breast cancer cell line datasets related to hypoxia, they form finer sub-clusters in real clinical data.

### Transcription factor analysis reveals multiple potential mechanisms of regulation for gene sub-clusters

We have used the Enrichr analysis tool [45, 46] to identify the transcription factors (TFs) or histone modifiers which have been identified by the ChEA database [47] as potential regulators for significant numbers of genes in each one of the clusters, sub-clusters, and signatures that we have considered in this study. The ChEA database of TF regulation was inferred by from various genome-wide ChIP-chip, ChIP-seq, ChIP-PET, and DamID datasets [47]. The detailed results are shown in Additional file 7, while the TFs with adjusted combined scores (CS) exceeding 5.0 are listed in Table 6.

**Table 6:** TFs with significant enrichment in the clusters C1 and C2 as well as their sub-clusters

Cluster	TF	Adjusted CS^a^	Number of genes	Cluster	TF	Adjusted CS^a^	Number of genes
C1			504	C2			598
	MYC	7.9	217		HIF1A	9.4	70
	EKLF	7.5	118	C2a			204
	JARID1A	6.7	146	C2b			88
	ETS1	5.9	115		HIF1A	9.7	16
C1a			116	C2c			168
	KDM5B	8.1	70		HIF1A	9.2	24
	MYC	6.7	58	51-gene sig.^b^			51
C1b			273		HIF1A	17.2	17
	MYC	10.1	125	20-gene sig.^b^			20
	EKLF	9.0	77		HIF1A	10.1	8
	JARID1A	9.0	99		TRIM28	6.2	3
	ETS1	8.2	80				
	HOXC9	5.6	75				
	GABP	5.3	87				

^a^The adjusted combined score (CS) calculation is detailed in Additional file 7 where the complete Table is. Only TFs with adjusted CS higher than the threshold of 5.0 are included here

^b^The 51-gene and the 20-gene signatures are hypoxia-induced signatures identified in [5] and [24] respectively

The TF with the most enriched targets in C1 was found to be MYC, which are also significantly enriched in both of C1’s sub-clusters. MYC is an oncogene encoding a transcription factor which selectively amplifies genes that contribute to cell growth and proliferation [48]. In normal cells, MYC is only activated if both sufficient nutrients and growth signals were available, and is deactivated by active checkpoints or differentiation. In contrast, abnormal activation of MYC was seen in cancerous cells leading to unconstraint growth and proliferation while ignoring checkpoints [48]. These known facts about MYC highly agree with the biological processes and pathways enriched in C1, C1a, and C1b, which are mainly related to cellular growth and proliferation.

In C2the solo significant player is HIF1A, which needs no further justification. However, C2a is not as significantly enriched with the targets of HIF1A as its parent cluster and two sibling sub-clusters do. This is consistent with the fact that it is not correlated with the hypoxia-response subset C2b in clinical datasets. Nonetheless, the top TFs in C2a are HIF1A, VDR, KDM5B, and FOXM1 with the respective adjusted CS values of 3.1, 3.1, 3.0, and 3.0. Notably, VDR and KDM5B appear in either or both of the C1 sub-clusters, either with a greater value than the threshold or just under it. These two TFs do not appear in the other two C2 sub-clusters (C2b and C2c) with any adjusted CS values close to significant.

### Sub-clusters of hypoxia co-regulated genes are associated with poor prognosis of tumours and are correlated with estrogen receptor status

We have investigated the prognostic significance, measured by hazard ratios (HR), and the relation with estrogen (ER) of the sub-clusters of C1 and C2 in the five clinical datasets listed in Table 5 which have overall survival (OS) and ER status information. In order to compare our sub-clusters with the state-of-the-art hypoxia signatures, we have also applied the same analysis to a signature of 51 genes previously developed using large meta-analysis of multiple datasets from different cancers [5], which was shown to be more robust than other hypoxia signatures [6], and to the consensus group of 20 genes recently compiled as the most frequently appearing genes across 32 different hypoxia signatures [24]. We shall refer to these two signatures hereinafter as the 51-gene signature and the 20-gene signature.

C2 has significant overlaps with the 51-gene and the 20-gene signatures, as it includes 19 and 12 of their genes, respectively. On the other hand, C1 does not have any significant overlaps with them. This is expected as C1 is down-regulated while the 51- and 20-gene signatures are upregulated under hypoxia. Looking into the sub-clusters of C2, C2b (88 genes) has the largest overlap with those two signatures as it includes 6 and 4 of their genes, respectively.

**Fig. 7:**
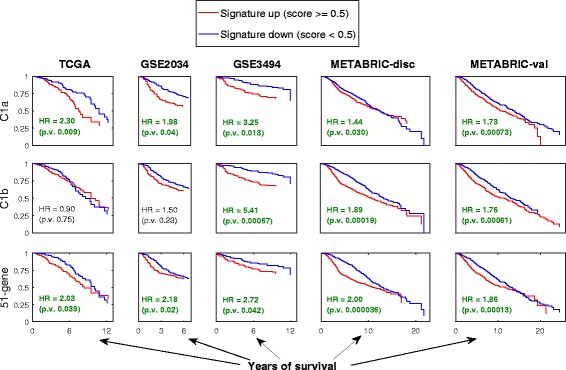
Survival curves for sub-clusters and signatures in clinical datasets. Survival curves for the sub-clusters C1a and C1b, as well as the 51-gene signature based on the five clinical datasets TCGA, GSE2034, GSE3494, METABRIC-disc, and METABRIC-val. In each sub-plot, the vertical axis represents the survival rate (probability of survival) while the horizontal axis represents time in years. The hazard ratios (HR) and the associated *p*-values are shown on the plots while highlighting those with significant *p*-values (<0.05) with boldface green font. Additional file 8 shows a complete version of this Figure including all other sub-clusters/signatures (C2a, C2b, C2c, and 20-gene). None of these other sub-clusters/signatures shows significant *p*-values in more than one out of five datasets

Figure 7 shows the survival curves of the sub-clusters C1a and C1b as well as the 51-gene signature. The Figure shows their hazard ratios (HR) and the associated *p*-values as estimated by Cox proportional hazards regression based on the patients’ overall survival time (OS). Entries with *p*-values smaller than 0.05 are considered significant and are marked with a boldface green font in this Figure. Indeed, higher HR values (HR > 1.0) indicate bad prognosis while lower HR values (HR < 1.0) indicate good prognosis. The clusters or signatures which have significant HR values consistently over all five clinical datasets are C1a (116 genes) and the 51-gene signature; both suggest bad prognosis (HR > 1.0). Moreover, C1b also shows significant bad prognostic values, but in three datasets only. The rest of the sub-clusters/signatures do not show significant *p*-values in more than a single dataset, and their survival curves are shown in Additional file 8.

**Fig. 8:**
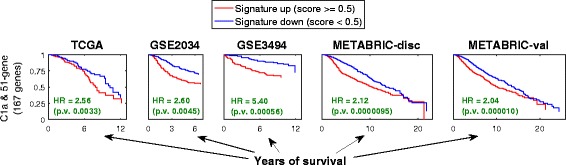
Survival curves for a combined signature composed of C1a and the 51-gene signature. The survival curves are based on each one of the five clinical datasets. The HR values and their associated *p*-values are shown

As the upregulation of both C1a and the 51-gene signature in clinical data indicate bad prognosis, we have calculated HR values for a combined signature composed of both of them. This combined signature’s survival curves and HR values are shown in Fig. 8. It is clear in this Figure that the combined signature shows higher HR values and more significant *p*-values than each one of the two signatures separately in each one of the five datasets. This indicates that the co-upregulation of both of those hypoxia-induced genes and those MYC targets is a stronger indication of bad prognosis than any of them independently.

**Fig. 9:**
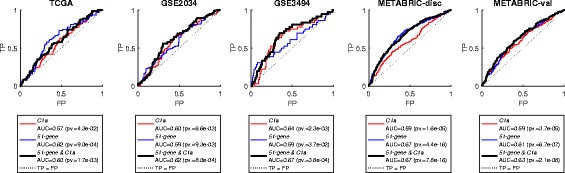
ROC curves for the signatures C1a and 51-gene separately and combined over the five clinical datasets. The combined signature is shown in a thicker line. Moreover, the area under the curve (AUC) for each of these curves as well as the associated *p*-values are shown in the legends below the plots

We further investigated this prognostic power by plotting the receiver operating characteristic (ROC) curves of true positives (TP) versus false positives (FP) for C1a and the 51-gene signature as well as their combination (Fig. 9). It is clear that the signatures C1a and 51-gene have consistent significance of prognostic power over the five datasets when considered individually (*p*-value <0.05). However, neither outperforms the other in all of the datasets. Interestingly, when they are combined, their prognostic power clearly improves, and either outperforms both individual signatures or is very close to the better one amongst them in all five datasets. Again, this shows that the conjoint up-regulation of these two subsets of genes in clinical tumours, which are regulated oppositely under hypoxia in vitro, is a strong indication of poor prognosis.

Table 7 shows expression fold-changes of the sub-clusters and signatures in ER^+^ samples versus ER^−^ samples (ER^+^/ER^−^) as estimated by analysis of variance (ANOVA). All sub-clusters of C1 and C2 and the 51-gene signature have significant fold-changes except for C2c. Interestingly, C2a goes up in ER^+^ samples (fold-change >1), while C1a, C1b, C2b, and the 51-gene signature go down (fold-change <1). The fact that C2b and the 51-gene signature do so, confirms ER+ being less hypoxic than ER^−^ tumours as discussed previously.

**Table 7:** Fold-changes of expression of signatures in relation to ER (ER^+^/ER^−^) and their associated *p*-values between parentheses

Sub-cluster/signature	TCGA	GSE2034	GSE3494	METABRIC-disc	METABRIC-val
C1a	**0.88 (0.006)**	**0.94 (0.033)**	**0.94 (7.5 × 10** ^**−4**^ **)**	**0.95 (1.1 × 10** ^**−4**^ **)**	**0.94 (1.5 × 10** ^**−6**^ **)**
C1b	**0.77 (8.8 × 10** ^**−18**^ **)**	**0.89 (1.6 × 10** ^**−11**^ **)**	**0.91 (4.9 × 10** ^**−16**^ **)**	**0.91 (2.0 × 10** ^**−29**^ **)**	**0.90 (1.3 × 10** ^**−38**^ **)**
C2a	**1.28 (8.3 × 10** ^**−13**^ **)**	**1.14 (2.1 × 10** ^**−10**^ **)**	**1.10 (1.9 × 10** ^**−12**^ **)**	**1.08 (1.3 × 10** ^**−17**^ **)**	**1.09 (4.1 × 10** ^**−20**^ **)**
C2b	**0.56 (4.3 × 10** ^**−28**^ **)**	**0.74 (1.0 × 10** ^**−23**^ **)**	**0.86 (1.3 × 10** ^**−14**^ **)**	**0.79 (1.9 × 10** ^**−59**^ **)**	**0.81 (4.4 × 10** ^**−51**^ **)**
C2c	1.01 (0.78)	1.01 (0.53)	1.02 (0.16)	**1.02 (0.025)**	**1.04 (5.4 × 10** ^**−4**^ **)**
51-gene signature	**0.63 (1.4 × 10** ^**−10**^ **)**	**0.76 (1.8 × 10** ^**−12**^ **)**	**0.86 (7.0 × 10** ^**−9**^ **)**	**0.80 (2.0 × 10** ^**−30**^ **)**	**0.81 (1.7 × 10** ^**−26**^ **)**
20-gene signature	**0.71 (0.003)**	**0.81 (8.6 × 10** ^**−4**^ **)**	0.96 (0.35)	**0.86 (3.4 × 10** ^**−7**^ **)**	**0.90 (2.6 × 10** ^**−4**^ **)**

Significant fold-changes with *p*-values smaller than 0.05 are shown in boldface

We then investigated if the OS times of the samples with the ER^+^ status and the ones with the ER^−^ status differ significantly by applying ANOVA analysis, which is the type of statistical analysis to assess if two groups of values are significantly different. Results showed that there was no significant differential OS time between ER^+^ and ER^−^ samples in three out of five clinical datasets (*p*-values: 0.42 in TCGA, 0.20 in GSE2034, 0.26 in GSE3494, 6.1 × 10^−4^ in METABRIC-disc, and 2.6 × 10^−6^ in METABRIC-val).

**Fig. 10:**
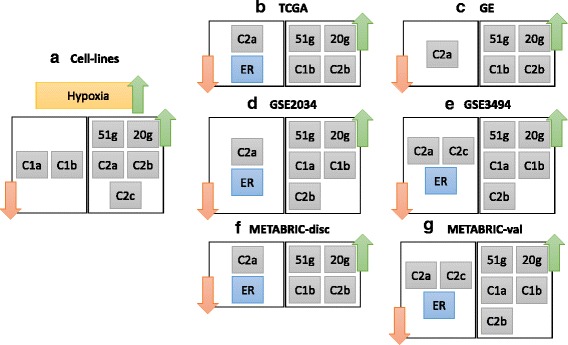
Expression relations amongst the sub-clusters and signatures in cell line and clinical datasets. Part **a** shows that in the 16 cell-line datasets, when hypoxia occurs, C1 sub-clusters are repressed while C2 sub-clusters are up-regulated. The 51-gene and the 20-gene signatures were previously established as signatures of hypoxia, and they are indeed up-regulated under hypoxia. Parts **b** to (**g**) show the gene expression relations amongst the different sub-clusters or signatures in each one of the six considered clinical datasets. Also, the ER status is included where its information is available. For example, **b** shows that in the TCGA dataset, when ER is down (negative), C2a genes go down in expression, while the expression of the genes in C1b, C2b, and the 51-gene and 20-gene signatures go up

Pairwise Spearman’s correlation amongst the summarised expression profiles of the sub-clusters and the signatures in the six clinical datasets has been calculated (Additional file 9). The summarised expression profile of a signature or a cluster is represented by its hypoxia scores (HS) as detailed in Methods. The results are summarised in Fig. 10. Interestingly, the Figure shows that the two sub-clusters of C2 (C2a and C2b), which are consistently correlated in cell line datasets, completely lose their correlation in all six clinical datasets. On the other hand, C1b joins C2b in having consistent positive correlation with the 51-gene signature in each one of the six datasets (*ρ* ranging from 0.35 and 0.6; Fig. 10 and Additional file 9). It was expected for C2b to have such correlated profiles due to its overlap with the 51-gene signature, but C1b does not have any significant overlap with the signature in terms of its gene-content. Indeed, C1b, C2b, and the 51-gene signature are upregulated in ER^−^ samples (Table 7). Moreover, C1b and C2a, which behave oppositely and significantly under different ER statuses, are also consistently negatively correlated in expression over the six datasets (*ρ* from −0.45 to −0.85; Fig. 10 and Additional file 9).

### Comparison of UNCLES with the iCluster method

The iCluster method has become a commonly used method for clustering in cancer research, especially for multiple datasets [25, 49]. Therefore, we have compared our method with it. We have applied iCluster to the same set of 16 cell-line datasets using its default parameters. The iCluster method requires pre-setting the number of clusters (*K*) to which the genes should be partitioned, so we applied it many times with *K* values ranging from 2 to 54. The details of the application of the method are provided in Additional file 10.

**Fig. 11:**
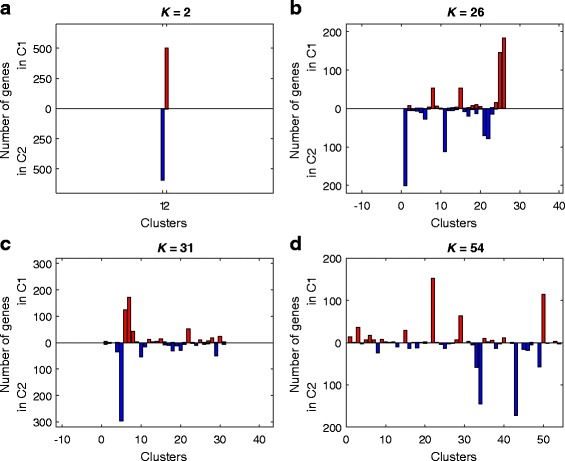
A comparison between the results of UNCLES and the results of iCluster. The distribution of the 504 genes in C1 and the 598 genes in C2 over the clusters generated by iCluster at **a** *K* = 2, **b** *K* = 26, **c** *K* = 31, and **d** *K* = 54. The horizontal axis in each plot represents the *K* clusters generated by iCluster in that case. For each iCluster cluster, i.e. at each point of the horizontal axes, two bins are shown, one upwards and one downwards. The upward bin reflects the number of genes from our C1 cluster that are included in that corresponding iCluster cluster, while the downward bin reflects the number of genes from our C2 cluster therein. A key observation is that none of the iCluster clusters shows large bins in both directions

One key difference between UNCLES and iCluster is that the iCluster method forces all of the 15,588 input genes to be included in one of its output clusters. For example, at *K* = 26, iCluster partitions the 15,588 genes into 26 clusters without filtering out any gene. This fact is important to remember while comparing the results of the two methods. Figure 11 shows the distribution of the genes included in our clusters C1 (504 genes) and C2 (598 genes) over the clusters generated by iCluster at four different *K* values, 2, 26, 31, and 54. Two major observations can be seen here; the first one is that the contents of our C1 and C2 clusters are always included in different iCluster clusters at any given *K* value; in other words, if one of the iCluster clusters includes a significant number of C1 genes, it does not include a significant number of C2 genes at the same time (no single point on the horizontal axes in Fig. 11 shows large upwards and downwards bins at the same time). This validates our UNCLES clusters as it is an evidence of their high separability. The second observation is that as iCluster includes the entire input set of genes (15,588) into clusters, it does not have a mechanism to select few clusters with the highest priority, and therefore does not imply filtering. For instance, at *K* = 2, iCluster partitions the 15,588 input genes into two clusters with 7317 and 8217 genes, respectively. Although each one of these two iCluster clusters exclusively includes one of our two UNCLES clusters entirely (Fig. 11 (A)), those two iCluster clusters are relatively giant and are much less specific than our UNCLES clusters.

## Discussion

We have demonstrated that poor prognosis can be predicted in breast cancer clinical samples by the conjoint up-regulation of two distinct subsets of genes which are rather observed to be oppositely regulated under hypoxia in sixteen in vitro breast cancer cell-line datasets. One of these two subsets of genes is upregulated under hypoxia in cell-lines and is enriched with HIF targets that have roles in response to hypoxia. In contrast, the other subset of genes is down-regulated under hypoxia in cell-lines and is enriched with MYC targets that are involved in various growth processes. Interestingly, the gene expression values of these two subsets are positively correlated in six examined clinical datasets, predicting poor prognosis when up-regulated, and demonstrating a clear case of deep disagreement between cell-lines and clinical data.

To be able to generalise our results within the context of breast cancer tissues under hypoxia, the 16 cell-line datasets that we analysed were chosen from that context but from various cell-lines and biological conditions (Table 1), and this is feasible due to the capability of the developed computational methods herein. Moreover, the microarray platforms of these datasets vary widely in terms of the manufacturer (e.g. Affymetrix and Illumina) and the actual model. This allows for the inaccuracies and missing data which may exist in some datasets to be overcome by the majority of the other datasets. In other words, although every platform has its own inherent imperfections, they are reduced by using the entirety of the 16 datasets.

The core results have been obtained by applying a clustering approach which we have developed recently [26] and improved in this study, and which can be employed to identify cohorts of co-regulated genes from collections of many datasets generated under diverse conditions. Comparisons with the commonly used iCluster method have revealed the unique ability of our approach to analyse integrated data at such a scale.

### What causes this mismatch between in vitro cell-lines and clinical data?

The mismatched relation between hypoxia-repressed genes (C1a) and hypoxia-induced genes (C2b and the 51-gene sub-cluster) in cell-line data versus clinical data may be due to their transcriptional regulators. Results showed that MYC seems to be the main activator of C1 and its sub-clusters (C1a and C1b) while HIF1A is the main activator of C2 and two of its sub-clusters (C2b and C2c) (Table 6). HIF1A and MYC interplaying is well-known in cancers [10]; under hypoxia, HIF1A represses MYC through different suggested ways, such as activating MXI-1, which is an antagonist of MYC with regards to mitochondrial biogenesis [50, 51], or by competing with MYC in binding directly to MAX, without which MYC does not carry out its function [48], or by binding to Sp1 to result in the displacement of MYC from Sp1 complexes and consequently decreased MYC activity [52]. These facts well justify the consistent anti-correlation observed between these two groups of genes in cell lines.

In contrast, and in all six clinical datasets, either or both sub-clusters of genes activated by MYC (C1a and C1b) show positive correlation with those activated by HIF1A (mainly C2b) (Fig. 10 (b-g)). Apparently, in vivo further selection, maybe through mutations of genes like TP53 and HER2, or other micro-environmental or epigenetic factors, overcomes the suppression effect of hypoxia mediated by HIF on these MYC-regulated growth genes. The expression of MYC itself is significantly upregulated in ER^−^ samples versus ER^+^ samples in four out of five clinical datasets with ER information (*p*-values of 6 × 10^−8^, 5 × 10^−4^, 0.8, 1 × 10^−3^, and 9 × 10^−9^ respectively). Indeed, HIF1A is significantly upregulated in ER^−^ samples in all five datasets (*p*-values of 5 × 10^−17^, 5 × 10^−7^, 5 × 10^−4^, 5 × 10^−20^, and 9 × 10^−24^ respectively). Overexpression of MYC was shown to overcome the competition of HIF1A with it over MAX, and thus overexpressed MYC maintain stable MYC-MAX heterodimers despite the presence of HIF1A [53], and is associated with poor outcome [54]. Nonetheless, what causes MYC to be overexpressed in tumours and not in cell lines? Also, MYC does not seem to be overexpressed in ER^−^ tumours in one of the clinical datasets, yet its predicted targets involved in growth and proliferation are upregulated. Is there an alternative regulatory machinery which upregulates such a group of genes despite the presence of HIF1A and the absence of MYC?

For instance, HIF2A (EPAS1) was shown to have a promoting effect on MYC as opposed to HIF1A, and it was suggested that those two HIF factors are likely to negate each other’s effect on MYC when both are present [10, 53]. However, as seen in the expression of C1 and C2 in the dataset D13, knocking down HIF2A in the MCF7 cell line showed know difference in the expression of either C1 or C2 compared with wild-type cells, but knocking down HIF1A did down-regulate C2 and up-regulate C1, and that was similar between knocking down HIF1A only and a double-knocking down of both factors (Fig. 2 and Additional file 2). Also, there is no significant upregulation of HIF2A in ER^−^ samples versus ER^+^ in any of the clinical datasets (*p*-values always higher than 0.1).

Such results further demonstrate, as discussed in previous studies and reviews [24], that cell lines, despite their usefulness, do not accurately represent real clinical tumours. We provide the community with new significant signatures of hypoxia which are derived from cell lines, and then mapped to clinical samples to derive prognostic signatures, namely C2b and the more interesting C1a. We also provide such a thorough comparison between the gene expression of different groups of genes under clinical or cell line datasets, which may lead to a better understanding of the factors influencing the differences between the two systems.

### Speculations regarding novel association of genes with hypoxia response

A few genes with unknown or poorly understood functions have been found in the clusters C1 and C2. The most notable ones are RSBN1 (chromosomal location 1p13.2) and KIAA0195 (aka TMEM94; chromosomal location 17q25.1), which are induced under hypoxia, and they have been identified as potential HIF targets by 7 or 5 different HIF target lists respectively (Table 4).

Only one study focused on RSBN1 gene or on any of its homologues [55]. This study suggested that murine RSBN1 may have a transcriptional regulatory role in haploid germ of male mice as it specifically localise in the nucleus at stages VII-VIII of murine spermatogenesis [55]. Interestingly, scrutiny of Takahashi and colleagues’ experiments shows that they investigated RSBN1’s expression in the brain, heart, intestine, kidney, liver, lung, muscle, ovary, spleen, and testis of male mice, to conclude that it is only expressed in the testis [55]. In other words, expression of RSBN1 in murine female breast tissues was not investigated, and it may well be regulated by HIF as all seven lists include it, it may be expressed in breast tissues, and may have a role in response to hypoxia in breast cancer consequently.

Few genome-wide studies identified interactions between KIAA0195, or one of its homologues, and some other gene products, such as the kinase CDK5 [56–58], ubiquitin C (UBC) [59, 60], and the *Drosophila*’s CG9099 protein [61]. To find KIAA0195 consistently co-expressed with many other HIF targets in our study and in five out of seven lists of potential HIF targets indicates that this poorly understood gene may be a genuine target of HIF and may also have a role in response to hypoxia.

## Conclusions

Poor prognosis can be predicted by the conjoint up-regulation of two subsets of genes in breast tumours, which we identified in this study. The first subset is enriched with MYC targets participating in various growth-related processes, while the second subset is enriched with targets of the hypoxia-induced factor (HIF) and is expected to participate in response to hypoxia. The HIF-targets subset includes genes that are consistently co-expressed in sixteen different breast cancer cell-line datasets generated from conditions related to hypoxia. As expected, it shows up-regulation exclusively under hypoxic conditions in all of these cell-lines, which agrees with the literature associating hypoxia with bad prognosis. Strikingly, the MYC-targets growth-related subset shows an opposite profile in all tested cell-line datasets, where its genes are always repressed by hypoxia. This is despite the fact that its up-regulation in tumours indicates poor prognosis. The repression of growth-related genes under hypoxia agrees well with expectations, which we observe in cell-lines (in vitro). However, the association of their up-regulation in tumours (in vivo) with poor prognosis is an interesting finding. In fact, we showed that the conjoint in vivo up-regulation of both subsets combined with an existing state-of-the-art hypoxia signature is as strong in predicting bad tumour outcomes as that state-of-the-art signature and potentially stronger.

These results were found by analysing six clinical datasets after the application of the UNCLES method, with its novel and important extension that we introduced in this study, to sixteen different genome-wide gene expression datasets of breast cancer cell-lines. These datasets were generated by different groups, using different platforms, and under different conditions related to hypoxia. This method, which is publically available as part of the *Clust* python package [27, 62], can be applied to other sets of gene expression datasets from other areas in order to find those subsets of genes which are consistently co-expressed over all of them. The uniqueness of the ability to perform this task fruitfully at a genome-wide scale as such has been demonstrated by comparisons with iCluster, which is a relevant state-of-the-art method.

## Methods

### Uncles

The *unification of clustering results from multiple datasets using external specifications (UNCLES)* has two types, A and B. Type A mines a set of datasets for the subsets of genes which are consistently co-expressed in all of them. On the other hand, type B identifies the subsets of genes which are consistently co-expressed in a subset of datasets while being poorly co-expressed in another subset of datasets. Here, we adopt UNCLES type A, which is applied by applying the *binarisation of consensus partition matrices (Bi_CoPaM)* method. The Bi-CoPaM method consists of four main steps [27, 63, 64]:

1. Generation of many partitions for the same set of genes by applying *C* different clustering methods over the expression profiles of these genes from *L* different microarray datasets. As each of these *C* methods is applied independently to each one of the *L* microarray datasets, this step generates *R* = *C* × *L* different partitions. All of these partitions should have the same number of clusters (*K*).
2. Relabelling the *R* partitions. The clusters within each partition are permuted such that the *i* ^th^ cluster from one partition corresponds to the *i* ^th^ cluster from each one of the other partitions. This is essential because, due to the unsupervised nature of clustering, different partitions are not guaranteed to have this alignment of their clusters. We adopt the *min-min* relabelling technique [26] here because it aims at giving the fittest clusters a higher priority to be properly matched even if the poorer clusters are improperly matched. This is in contrast to the *min-max* technique [27] which aims at giving poorer clusters a chance to be fairly matched even if the compensation was to reduce the matching quality of the fittest clusters. The latter is better when all of the clusters are of interest, while the former is more suitable for the aim of our study, in which we prefer to optimise the fittest clusters and eliminate the remaining clusters from the final result.
3. Generation of the fuzzy consensus partition matrix (CoPaM) by element-by-element averaging of the relabelled partitions. This fuzzy CoPaM assigns each gene to each one of the *K* different clusters with a fuzzy value ranging from zero (does not belong) to unity (perfectly belongs).
4. Binarization of the CoPaM by one or more of the six tuneable binarization techniques proposed in [27]. Here we adopt the *difference threshold binarisation (DTB)* technique, which assigns a gene to the cluster in which it has the highest fuzzy membership value if, and only if, the fuzzy membership value of that gene in any of the other clusters is lower than that highest value at least by the value of the tuning parameter *δ*. In other words, if two or more clusters compete over a given gene with close fuzzy membership values, the gene is not considered distinctly belonging to a single specific cluster, and is, consequently, not assigned to any of them. The tightness of the clusters is therefore controlled by the value of *δ*, which ranges from zero (loosest) to unity (most stringent).

The UNCLES method is implemented as part of the open-source Python package “Clust” available on https://github.com/BaselAbujamous/clust [62].

### Extension of UNCLES

**Fig. 12:**
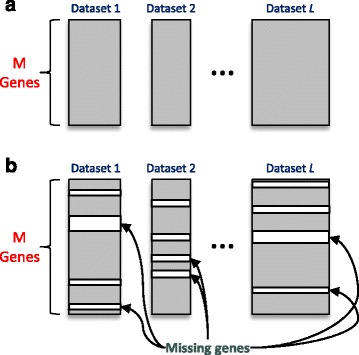
A demonstration of the difference between the original UNCLES and the extended one. **a** Original UNCLES: all datasets must have identical sets of genes. **b** Extended UNCLES: some datasets may miss some of the input set of genes. Both **a** and **b** allow datasets to have different numbers of samples

The UNCLES method, as was proposed in [26], requires that all of the datasets measure the expression profiles of identical sets of genes (Fig. 12 (A)). We propose a novel extension of the UNCLES method here to allow it to analyse multiple datasets where genes are included at least in *L* ^*^ out of the given *L* datasets. In this case, the individual clustering methods (e.g. *k*-means, SOMS, etc.) are applied to each dataset individually based on the genes which are represented by that dataset only. After that, when the individual results are combined by averaging element-by-element to produce the fuzzy CoPaM matrix, gene membership values are averaged only based on, and weighted by, the datasets which include that gene, even if not all of them.

This extension of the UNCLES method is implemented as part of the open-source Python package “Clust” available on https://github.com/BaselAbujamous/clust [62].

### M-N scatter plots technique

The UNCLES method requires a fixed number of clusters (*K*) to be set as well as the tuning parameter *δ*. However, the best values of *K* and *δ* may not be easily set manually. The M-N scatter plots technique addresses this issue by selecting the best clusters of the results of the application of UNCLES multiple times, each with a different *K* value and with varying *δ* values. All of the individual clusters from these different results of UNCLES are scattered on a 2-D plot. Each point on the plot represents one cluster, the horizontal axis represents the dispersion of the cluster measured by a modified version of the mean-square error (MSE) metric, and the vertical axis represents the logarithm of the size of the cluster, i.e. the logarithm of the number of genes included in the cluster.

Aiming at minimising the dispersion of the clusters (horizontal axis) while maximising their sizes (vertical axis), the cluster which is represented by closest point to the top-left corner of the plot is selected as the best cluster. This solves the problem of merely minimising the dispersion, as the dispersion of the trivially small clusters which include one or few genes is usually the minimum. In contrast, the M-N scatter plots favours larger clusters which maintain as low dispersion as possible.

It is likely that many of the repetitions of UNCLES have produced clusters with similar contents. Therefore, after selecting the top-left cluster in the M-N plot, all of those clusters with overlapping contents are removed from the plot. Out of the remaining clusters in the plot, the one which is closest to the top-left corner is selected as the second best cluster. The same steps are repeated until the M-N plots are empty or until the researcher is satisfied with the top few clusters obtained.

As can be seen, M-N plots do not select a whole partition and does not select the best *K* value or *δ* values. It rather selects the best clusters ordered in quality, which might have been generated by different *K* or *δ* values.

The M-N scatter plots technique is implemented as part of the open-source Python package “Clust” available on https://github.com/BaselAbujamous/clust [62].

### Data pre-processing

Prior to clustering, the one-colour datasets were normalised by quantile normalisation [65]. Then each gene’s expression profile was shifted and scaled to be zero-mean and unity standard deviation. The log-ratios of the two-colour datasets were zero-centred by subtracting genes’ log-ratios’ mean values [66]. In case of having multiple replicates per condition, the median value was taken. In the case of having multiple probes in the same dataset representing the same gene, the representative probe was considered as the one with the maximum coefficient of variation while at least a quarter of its samples exceed the first quartile of expression values in that dataset. If none of the probes representing a gene exceeds that quartile, the one with highest coefficient of variation of all of its probes was considered.

### Statistical analysis of clinical datasets

Given a clinical dataset, a single representative probe-set is first selected for each gene which has multiple probe-sets. This is done by taking the most variable probe-set, as measured by covariance, out of the probe-sets exceeding the 25th percentile expression in at least 25% of the samples. If no probe-set for that particular gene exceeds the aforementioned threshold, the most variable probe-set amongst all of the genes’ probe-sets is selected. The dataset is thereafter normalised by quantile normalisation.

To test the prognostic capability of a signature given a normalised dataset, the signature is first summarised by taking the median expression value of its genes at each sample. The median values are ranked over the samples and then scaled to the range of 0.0 to 1.0. Those [0.0, 1.0] values are called the hypoxia scores (HS) of the signature over each one of the samples. The HS values are submitted to Cox proportional hazards regression analysis to obtain their hazard ratios (HR) and the associated *p*-values.

If the estrogen (ER) status of the samples is provided with the clinical dataset, analysis of variance (ANOVA) is performed for each gene in the dataset in order to identify if it is differentially expressed between ER+ and ER- samples. A *p*-value and the fold-changes are therefore associated with each gene. The average of the expression values of each gene was shifted to zero and the standard deviation was scaled to unity before submission to ANOVA analysis. The negative logarithm of those ANOVA *p*-values followed an approximately lognormal distribution, while the fold-changes followed, as they were, a lognormal distribution. Therefore, to test if the genes of a given signature have significantly lower ANOVA *p*-values or higher fold-changes over the ER variable compared with the rest of the genome, another ANOVA analysis was adopted over the logarithm of the negative logarithm of the *p*-values, and over the logarithm of the fold-changes.

## Additional files

Additional file 1: List of the 15,588 genes used as input to the UNCLES clustering analysis and the 504 and 598 genes in C1 and C2, respectively. (XLSX 411 kb)

Additional file 2: This Figure shows the labelled average profiles of clusters C1 and C2. This is the same as Fig. 2 but where the labels of the horizontal axes are fully provided. (PDF 510 kb)

Additional file 3: GO term analysis of the cluster C1. (XLSX 33 kb)

Additional file 4: GO term analysis of the cluster C2. (XLSX 32 kb)

Additional file 5: Many Tables related to the analysis of the clusters C1 and C2 based on the clinical TCGA dataset. (XLSX 936 kb)

Additional file 6: This Figure shows histograms of the expression values of the genes in C1 and C2 based on the TCGA dataset. (PDF 23 kb)

Additional file 7: These Tables show the transcript factor analysis of the various clusters, sub-clusters, and hypoxia signatures. (XLSX 544 kb)

Additional file 8: This Figure shows the survival curves of all of the sub-clusters of C1 and C2 as well as the 51-gene and the 20-gene signatures. Figure 7 in the main manuscript includes the most significant part of this Supplementary Figure. (PDF 91 kb)

Additional file 9: These heat maps or Tables show the correlation amongst the different clusters, sub-clusters, and signatures based on each one of the four considered clinical datasets. (XLSX 19 kb)

Additional file 10: This text describes further comparisons between the UNCLES method and the iCluster method. (PDF 260 kb)
