# Supplementary material for: In vitro downregulated hypoxia transcriptome is associated with poor prognosis in breast cancer
Source: Mol Cancer. 2017 Jun 15;16:105. doi: 10.1186/s12943-017-0673-0 (PMC5472949; doi:10.1186/s12943-017-0673-0)
Supplement: Supplementary file 2 — This Figure shows the labelled average profiles of clusters C1 and C2. This is the same as Fig. 2 but where the labels of the horizontal axes are fully provided. (PDF 510 kb) [file 12943_2017_673_MOESM2_ESM.pdf]

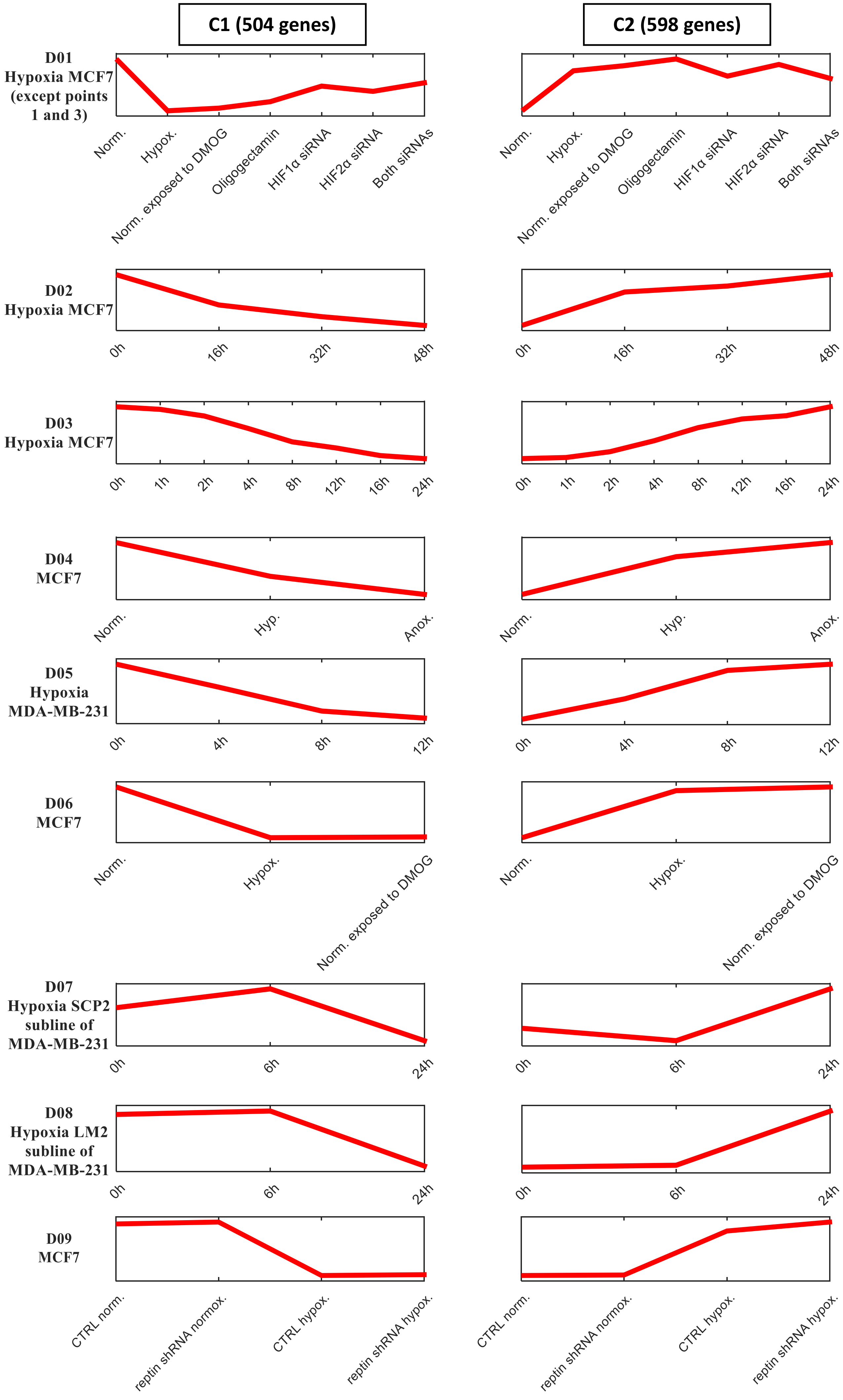
C1 (504 genes)
C2 (598 genes)
D01
Hypoxia MCF7
(except points
1 and 3)
Norm.
Hypox.
Norm. exposed to DMOG
Oligogectamin
HIF1α siRNA
HIF2α siRNA
Both siRNAs
D02
Hypoxia MCF7
0h
16h
32h
48h
D03
Hypoxia MCF7
0h
1h
2h
4h
8h
12h
16h
24h
D04
MCF7
Norm.
Hyp.
Anox.
D05
Hypoxia
MDA-MB-231
0h
4h
8h
12h
D06
MCF7
Norm.
Hypox.
Norm. exposed to DMOG
D07
Hypoxia SCP2
subline of
MDA-MB-231
0h
6h
24h
D08
Hypoxia LM2
subline of
MDA-MB-231
0h
6h
24h
D09
MCF7
CTRL norm.
reptin shRNA normox.
CTRL hypox.
reptin shRNA hypox.

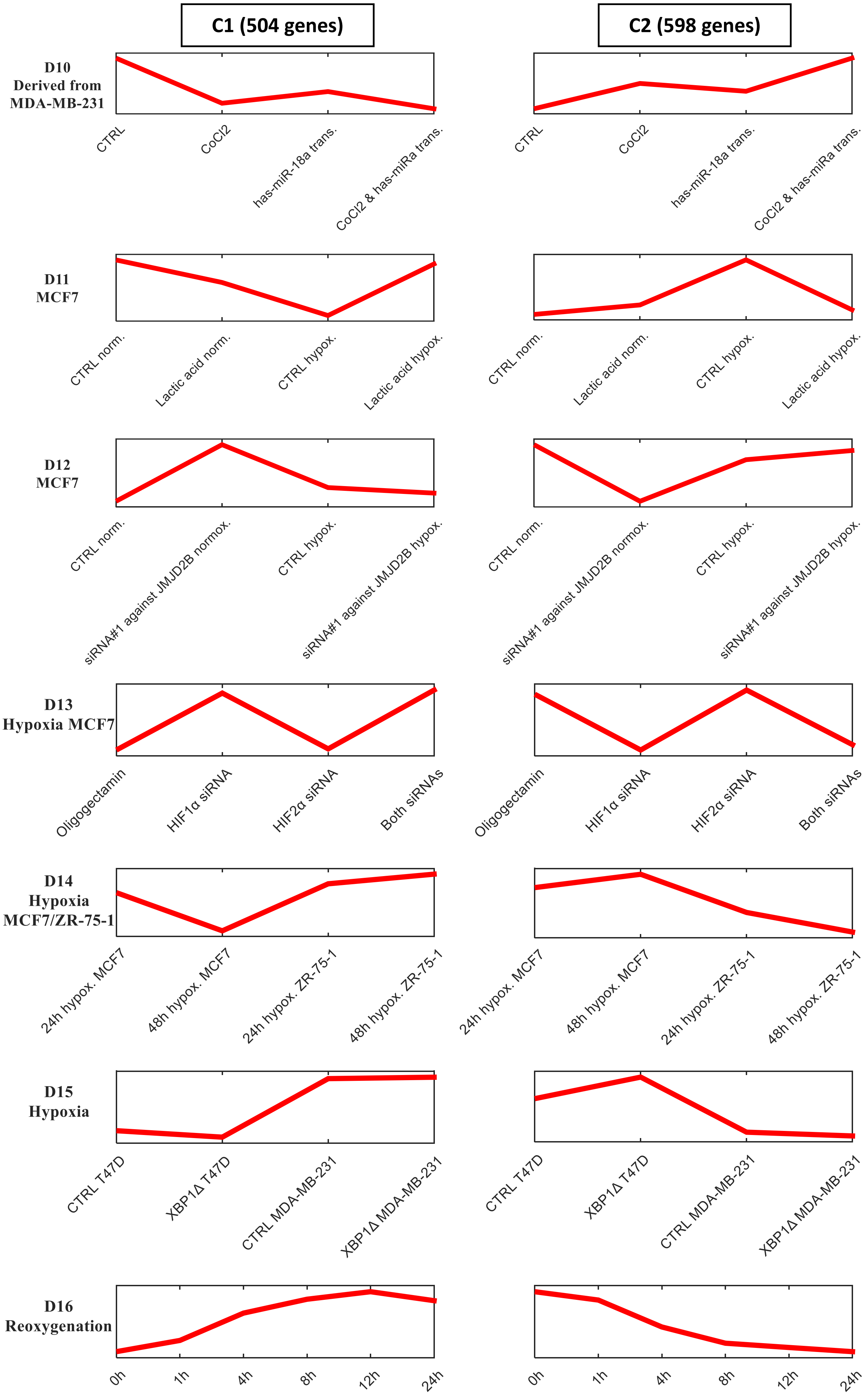
C1 (504 genes)
C2 (598 genes)
D10
Derived from
MDA-MB-231
CTRL
CoCl2
has-miR-18a trans.
CoCl2 & has-miRa trans.
D11
MCF7
CTRL norm.
Lactic acid norm.
CTRL hypox.
Lactic acid hypox.
D12
MCF7
CTRL norm.
siRNA#1 against JMJD2B normox.
CTRL hypox.
siRNA#1 against JMJD2B hypox.
D13
Hypoxia MCF7
Oligogectamin
HIF1α siRNA
HIF2α siRNA
Both siRNAs
D14
Hypoxia
MCF7/ZR-75-1
24h hypox. MCF7
48h hypox. MCF7
24h hypox. ZR-75-1
48h hypox. ZR-75-1
D15
Hypoxia
CTRL T47D
XBP1Δ T47D
CTRL MDA-MB-231
XBP1Δ MDA-MB-231
D16
Reoxygenation
0h
1h
4h
8h
12h
24h
