# Supplementary material for: In vitro downregulated hypoxia transcriptome is associated with poor prognosis in breast cancer
Source: Mol Cancer. 2017 Jun 15;16:105. doi: 10.1186/s12943-017-0673-0 (PMC5472949; doi:10.1186/s12943-017-0673-0)

Supplementary File S6

These are histograms for the log_2 expression values of the genes in the TCGA dataset over all of the 1026 tumour samples. The first histogram shows the expression values of all of the genes. This histograms has two modes; one mode is at the log_2 expression values of less than 4.0, including a very high bin at the expression value of 0.0, and represents unexpressed genes; the second mode is centred at about 9.5, and represents expressed genes. The second and the third histograms represent the expression values of the genes within the clusters C1 and C2, respectively, over the 1026 samples in the same TCGA dataset. It is clear that the genes within C1 and C2 are expressed in this clinical dataset as their values mainly fall within the second mode of the first histogram.

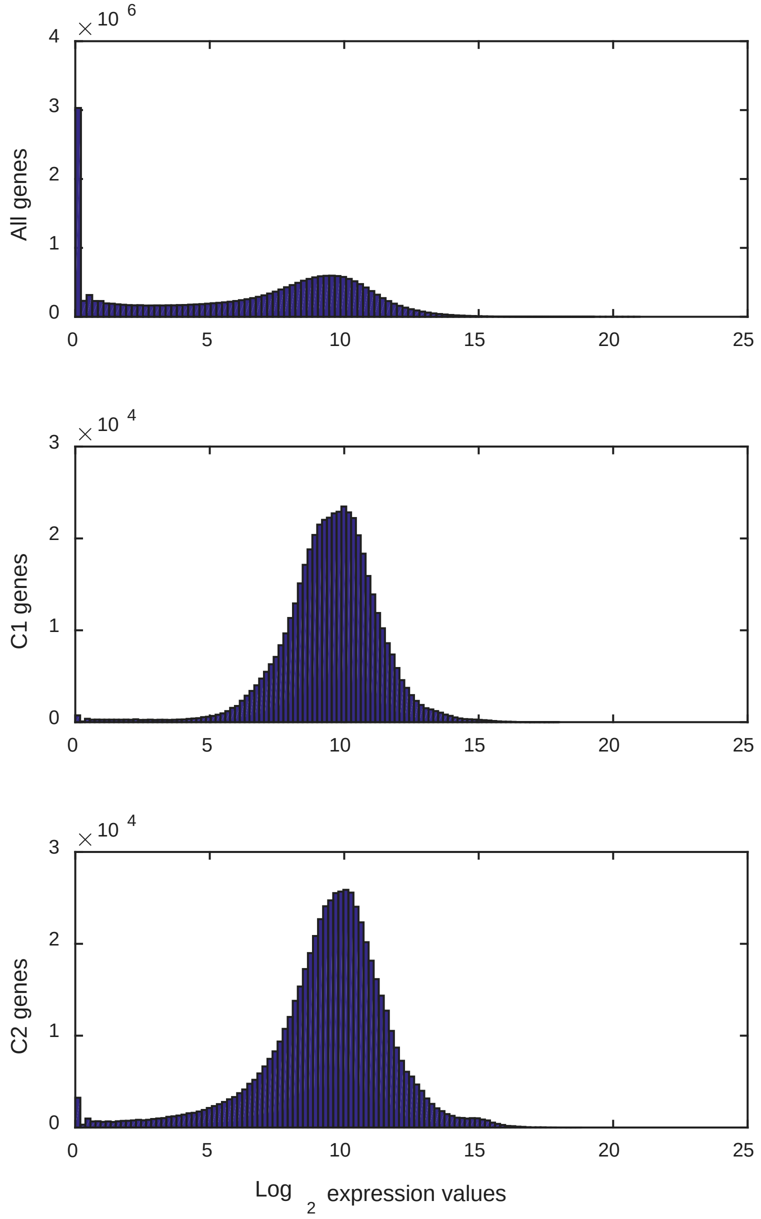

Supplement: Supplementary file 6 — This Figure shows histograms of the expression values of the genes in C1 and C2 based on the TCGA dataset. (PDF 23 kb) [file 12943_2017_673_MOESM6_ESM.pdf]
