# Supplementary figures and images for: In vitro downregulated hypoxia transcriptome is associated with poor prognosis in breast cancer
Source: Mol Cancer. 2017 Jun 15;16:105. doi: 10.1186/s12943-017-0673-0 (PMC5472949; doi:10.1186/s12943-017-0673-0)

— Signature up (score  $\geq 0.5$ )  
 — Signature down (score  $< 0.5$ )

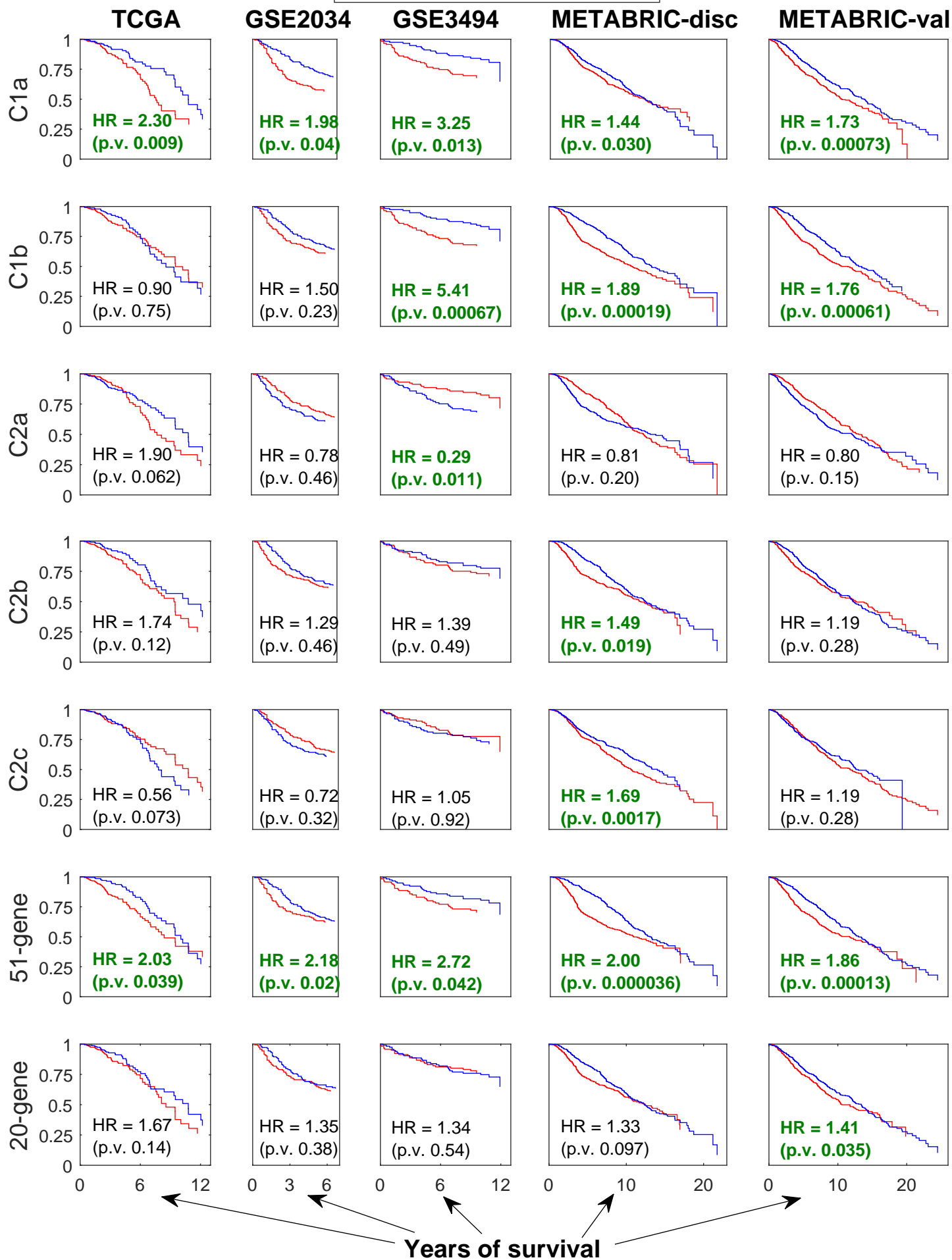

Supplement: Supplementary file 8 — This Figure shows the survival curves of all of the sub-clusters of C1 and C2 as well as the 51-gene and the 20-gene signatures. Figure 7 in the main manuscript includes the most significant part of this Supplementary Figure. (PDF 91 kb) [file 12943_2017_673_MOESM8_ESM.pdf]
