# Supplementary material for: In vitro downregulated hypoxia transcriptome is associated with poor prognosis in breast cancer
Source: Mol Cancer. 2017 Jun 15;16:105. doi: 10.1186/s12943-017-0673-0 (PMC5472949; doi:10.1186/s12943-017-0673-0)
Supplement: Supplementary file 10 — This text describes further comparisons between the UNCLES method and the iCluster method. (PDF 260 kb) [file 12943_2017_673_MOESM10_ESM.pdf]

Comparison with the iCluster method

The iCluster method, which is a joint latent variable model for integrative clustering, allows for the integrative analysis of datasets from different data types, such as gene expression data with copy number data from the same biological context [1,2]. We applied the latest version of this method as provided by the authors in an *R* package to those datasets using *K* values ranging from 2 to 54. The execution of that method failed to provide results for *K* values larger than 54 on this set of datasets. As iCluster cannot process datasets with different numbers of genes, we padded the missing genes in any dataset with zeros to allow the iCluster to run.

Moreover, and in compliance with the approach proposed by the founders of the iCluster method [1,2], we used their same *R* package to calculate the proportion of deviance (POD), which evaluates the separability of the clusters in a partition. Lower values of POD (closer to zero) indicate better separability while larger values (closer to one) indicate worse separability. Therefore, POD should be minimised for a higher quality result. The POD values for the clustering results using the aforementioned *K* values are shown in Figure 1.

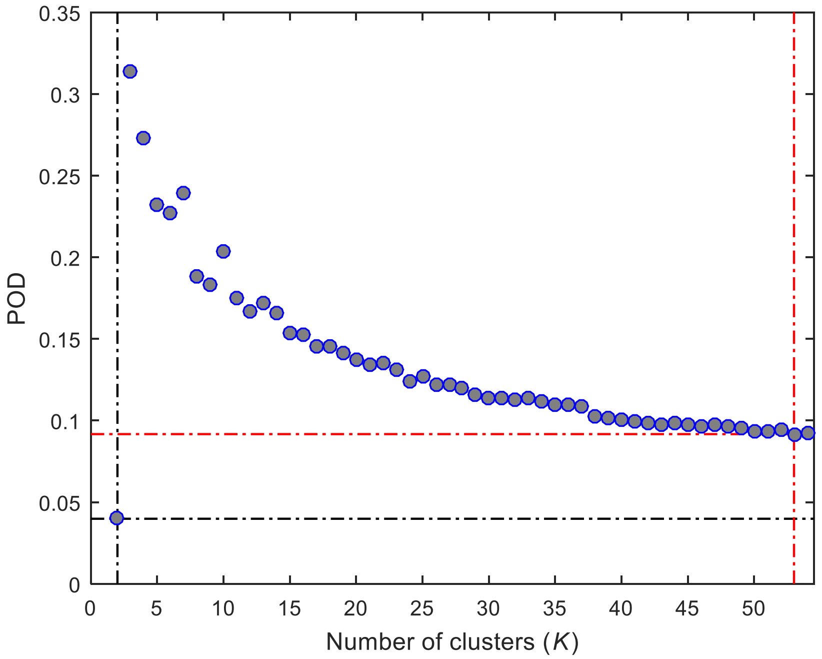

Figure 1. POD values for the results of iCluster application to the 16 datasets using *K* values ranging from 2 to 54. Lower POD values indicate better cluster separability. The lowest POD value is 0.04 at *K* = 2, and the second lowest POD value is 0.092 at *K* = 53.

As seen in this Figure, the lowest POD value (0.04) has been found at *K* = 2, partitioning the dataset into two giant clusters including 7,371 and 8,217 genes, respectively. The next lowest POD, which is much higher in value (0.092), has been found at *K* = 53. The average size of the clusters at this *K* value is 294 genes per cluster. Figure 2 shows the sizes of the clusters generated by iCluster at selected *K* values in the form of bar plots.

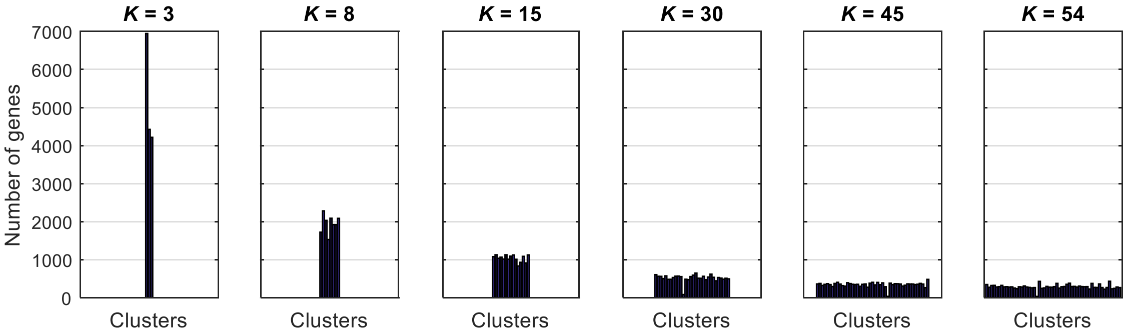

Figure 2. Bar plots showing the numbers of genes in each cluster generated by iCluster at selected *K* values. Each bar represents one cluster, while the vertical axis represents the size of the cluster. The vertical axis range of all of the six sub-plots is the same.

As can be seen in this Figure, the 15,588 genes in the input set are distributed over relatively similarly sized clusters in each one of these cases, which causes clusters to be smaller at higher values of *K*. Therefore, had the *iCluster2* method of that *R* package converged while clustering those datasets into *K* values greater than 54, it is most likely that the resulting clusters would have been similarly sized with sizes smaller than that at *K* = 54. Having said that, although the trend in Figure 1 predicts smaller POD values for untested greater *K* values, the trend in Figure 2 predicts smaller sizes of the clusters generated at such greater *K* values. Given that the sizes of the clusters at *K* = 54 are already too small, or at least much smaller than our C1 (504 genes) and C2 (598 genes), it is most likely that those iCluster results at high *K* values are not significant. This addresses the potential argument that the minimum POD value, other than the one at the special case of *K* = 2, has been found at the upper limit of the tested *K* values, which does not guarantee that it is the global optimum.

A major difference between our approach and iCluster is that our results include few clusters of genes in a descendant order of quality, while iCluster's results, at any given *K* value, include the entire input set of genes partitioned into many clusters, without any particular indication to particular clusters which deserve more focus. However, we take a step further and compare our clusters C1 and C2 with the clusters produced by iCluster at *K* = 2 and *K* = 54. For instance, each one of the two giant clusters generated by iCluster at *K* = 2 includes one of our two clusters (C1 and C2), almost entirely (503 out of 504 genes from

C1 genes are in one cluster, and 594 out of 598 genes from C2 are in the other cluster); see Figure 3 (A). In other words, those two giant clusters represent very wide versions of C1 and C2 in a way that encompasses the entire input set of 15,588 genes.

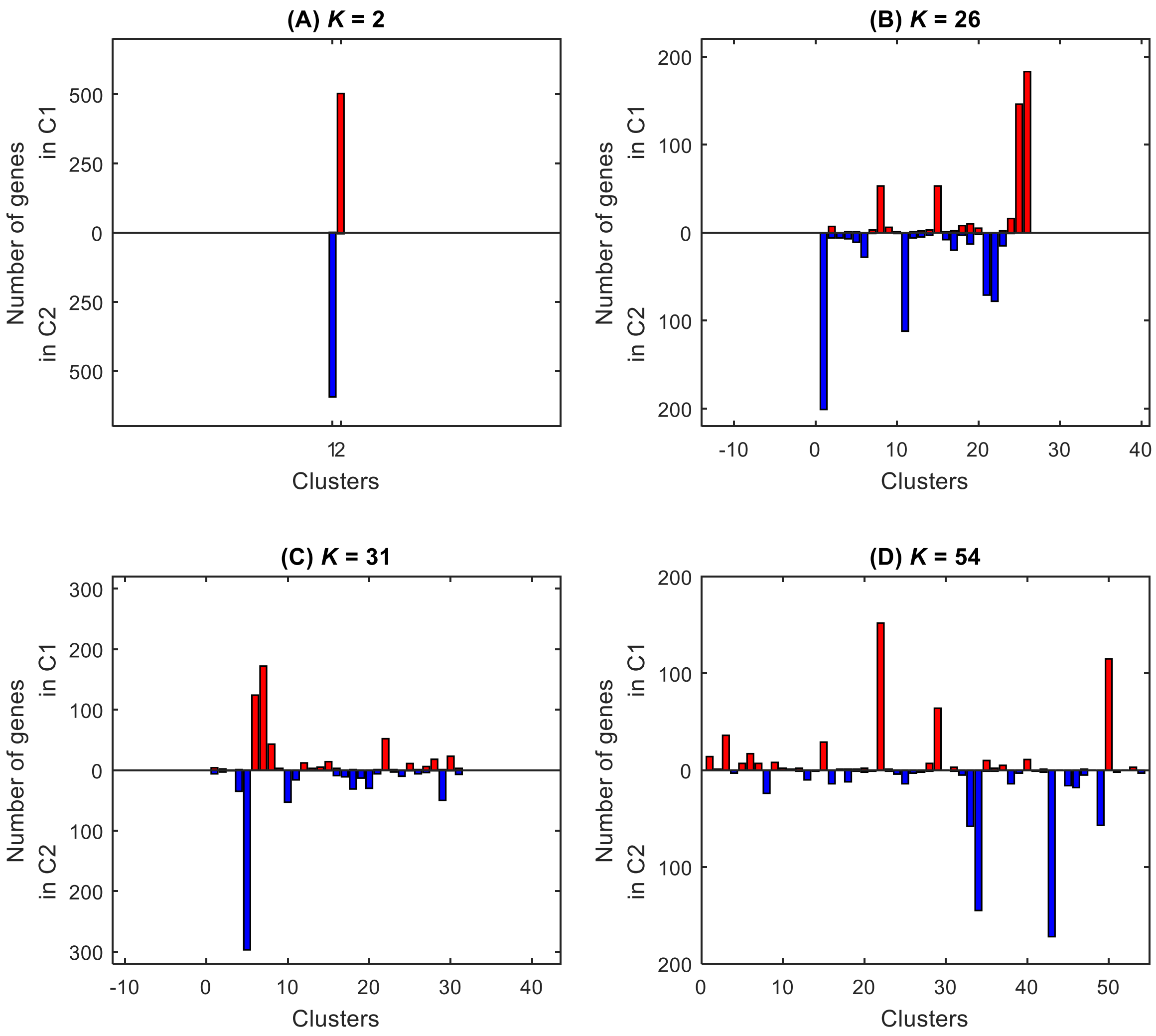

Figure 3. The distribution of the 504 genes in C1 and the 598 genes in C2 over the clusters generated by iCluster at (A) *K* = 2, (B) *K* = 26, (C) *K* = 31, and (D) *K* = 54. *K* values in (A) and (D) are the maximum and the minimum used ones and they resulted in relatively lower POD values, while the average sizes of the clusters generated at the *K* values in (B) and (C) are 600 and 503, respectively, which are the closest sizes to our C2 and C1, respectively.

On the other hand, the genes in our clusters C1 and C2 are distributed over various clusters in the results of iCluster at *K* = 54, as shown in Figure 3 (D). It is interesting that if one of the 54 clusters includes many C1 genes, it does not include many C2 genes, and vice versa. This agrees with our results as the genes in C1 and C2 are negatively correlated and are highly expected not to belong to the same cluster by any clustering method. A similar comparison has been conducted for the iCluster results generated at *K* =

26 and $K = 31$ (Figure 3 (B) and (C)), because the respective average sizes of the clusters at these results are 600 and 503, which are the closest cluster sizes to our C2 and C1 clusters, respectively. Again, the contents of C1 and C2 are distributed over many clusters at these K values without any of them being dominantly contained by a single cluster.

Taken together, iCluster analysis always includes the entire set of input genes in some clusters, which does not result in focused and ordered clusters as the UNCLES method does. Also, the POD index has pointed out that the results at $K = 2$ and, potentially, at $K = 54$, have the best separability results. Nonetheless, the results of our UNCLES method, namely the clusters C1 and C2, have not been found dominating any of the clusters at any of these K values, and not even at those K values which produce clusters of similar sizes to C1 and C2.

References

1. Shen R, Olshen AB, Ladanyi M: **Integrative clustering of multiple genomic data types using a joint latent variable model with application to breast and lung cancer subtype analysis.** *Bioinformatics* 2009, **25**:2906–2912.

2. Shen R, Mo Q, Schultz N, Seshan VE, Olshen AB, Huse J, Ladanyi M, Sander C: **Integrative subtype discovery in glioblastoma using iCluster.** *PLOS ONE* 2012, **7**:e35236.
